# Burden of chickenpox complications in Poland, 2006 to 2021: A comprehensive registry-based study

**DOI:** 10.2807/1560-7917.ES.2024.29.9.2300355

**Published:** 2024-02-29

**Authors:** Rafał Halik, Iwona Paradowska-Stankiewicz, Aneta Trochonowicz, Swavik Dittmer

**Affiliations:** 1 Department of Population Health Monitoring and Analysis, National Institute of Public Health NIH—National Research Institute, Warsaw, Poland; 2 Infectious Disease Epidemiology and Surveillance Department, Vaccine Preventable Diseases Unit, National Institute of Public Health NIH—National Research Institute, Warsaw, Poland; 3 NHS National Services Scotland, Digital and Security, Edinburgh, United Kingdom

**Keywords:** chickenpox, chickenpox complications, surveillance, chickenpox prevention

## Abstract

**Background:**

Chickenpox, a vaccine-preventable disease caused by the varicella zoster virus, generally presents with mild symptoms but can cause complications necessitating hospitalisation. In Poland, since 2009, vaccination has been obligatory for children up to 12 years of age who are particularly vulnerable to infection and for children in their vicinity.

**Aim:**

To examine the burden of chickenpox complications and the trends of hospitalisation arising from these complications over time in the Polish population.

**Methods:**

Data spanning 2006–21 were sourced from the Polish Infectious Diseases Surveillance System, the Nationwide General Hospital Morbidity Study and the Statistics Poland death registry. Standardised and age-specific incidence rates, hospital discharge rates and number of deaths because of chickenpox were calculated. Moreover, the joinpoint regression model was used to analyse trends of annual hospital discharge rates.

**Results:**

Over the analysed timeframe, 25,804 hospitalisations and 52 deaths attributable to chickenpox complications were documented, and 1.0% of chickenpox cases required hospitalisation because of chickenpox. Age-standardised hospitalisation rates varied between 2.3 and 9.6 per 100,000 population. The analysis revealed no statistically significant trend in overall hospital discharge rates from chickenpox complications. However, a notable increase in hospitalisation rates was observed in children aged 0–4 and among inhabitants of rural areas, with annual percentage changes of 4.9% and 3.4% respectively.

**Conclusions:**

Our findings suggest that the implementation of a universal chickenpox immunisation programme, supported by health education, should be considered to reduce the number of hospitalisations and nearly eliminate deaths because of chickenpox.

**Key public health message**

**What did you want to address in this study and why?**

Chickenpox, while usually a mild disease, can cause serious complications or death. In Poland, vaccination is only obligatory for children who are particularly vulnerable to infection. As there is little evidence on the burden of chickenpox complications observed in Polish general population, nor on the impact of immunisation to reducing chickenpox complications, we wanted to understand the trends of hospitalisation and deaths from chickenpox over time.

**What have we learnt from this study?**

Between 2006 and 2019, hospitalisation rates from chickenpox increased in children 0–4 years and in rural areas. The risk of chickenpox complications requiring hospitalisation in Poland seems to be higher than in other European Union countries. Merging data from routine surveillance and different medical registries can be a helpful tool to monitor the frequency of chickenpox complications.

**What are the implications of the findings for public health?**

Our findings suggest that chickenpox immunisation should be considered for all children in Poland, supported by health education and improvements in primary health care. Such intervention could reduce hospitalisations and nearly eliminate deaths in children with chickenpox complications by 95%.

## Introduction

Chickenpox (varicella) is a highly infectious viral disease caused by the varicella zoster virus (VZV) that has an estimated basic reproduction number (R_0_) ranging from 3.3 to 16.9 [1]. The primary symptoms of chickenpox include fever and rash that evolves into small, fluid-filled blisters. Infection is primarily transmitted through direct contact with fluid from the blisters or respiratory droplets from an infected individual. While the majority of cases, predominantly affecting children and adolescents, are mild and resolve without complications within 5–10 days, chickenpox can also lead to serious complications requiring hospitalisation. The most common complications include secondary purulent skin infection because of bacterial superinfection of chickenpox lesions, viral and bacterial pneumonia, aseptic encephalitis with cerebellar ataxia, and Reye's syndrome [2-4]. Neurological complications such as encephalitis and meningitis are relatively rare. According to the World Health Organization (WHO), each year there are 14 million complicated cases of chickenpox globally, resulting in ca 4,200 deaths [5]. 

Immunisation can greatly reduce the number of chickenpox complications by over 95% [6,7]. However, it is important to consider the cost-effectiveness of such vaccination programmes [8]. Poland authorised chickenpox vaccines in 1999 [9], and since 2003, vaccinations have been authorised and recommended for individuals who have not contracted chickenpox, and a physician could prescribe the vaccine after medical assessment. Furthermore, since 2009, vaccinations have been obligatory for children up to 12 years of age who are particularly vulnerable to infection, i.e. under care of orphanages, and for children in their vicinity [10]. Vaccines are free of charge and reimbursed from public funds. Data from the Polish Sanitary Inspection’s reporting system show that between 2006 and 2021, a total of 855,711 vaccine doses were administered [11], resulting in an estimated maximum vaccine coverage of up to 2.3% of the total Polish population in 2021. 

A Polish observational study conducted from 2010 to 2015 on a small cohort of hospitalised children identified the most common complications from chickenpox as dehydration (15.9%), skin and soft tissue infections (14.6%), pneumonia (12.2%), and cerebellitis (11.0%) [12]. However, there is little evidence on the overall burden of chickenpox complications in the Polish general population, nor are there observations examining whether introducing immunisation has had an impact on reducing chickenpox complications. Here, we examine the burden of chickenpox complications and the trends of hospitalisation given these complications over time from 2006 to 2021 in the Polish population in order to inform future interventions, including the intensification of immunisation efforts.

## Methods

### Data sources

We analysed all available data from sources collecting epidemiological characteristics of chickenpox cases in Poland from 2006 to 2021.

#### Incidence

The Polish Infectious Diseases Surveillance System (PIDSS) performs data collection of notifiable infectious diseases epidemiology. According to the Act of 5 December 2008 on preventing and controlling infections and infectious diseases in humans [13], chickenpox is one of the mandatory notifiable diseases in Poland, requiring reporting of infections by physicians to the Polish State Sanitary Inspection. Reported cases are verified, collected and registered by the Sanitary Inspection. Thereafter, data are also transferred to the National Institute of Public Health National Institute of Hygiene-National Research Institute (NIPH NIH-NRI), which performs epidemiological analysis and data aggregation. Data include demographic characteristics of the patient, place of residence and information on whether a course of infection required hospital treatment without specifying the exact causes of hospital discharge. The PIDSS applies International Statistical Classification of Diseases and Related Health Problems (ICD)-10 classification [14] and follows the standards of Statistics Poland in terms of demographic characteristics, and thus it means that variables are consistent with other data sources within public statistics in Poland. For reported varicella cases, the quality of data is high and missing data exist in approximately 4% of records.

#### Hospital morbidity

The Nationwide General Hospital Morbidity Study (NGHMS) is conducted by the NIPH NIH-NRI within the framework of public statistics [15]. The NGHMS collects data related to all cases of hospitalisation in Poland including the course of treatment and causes of hospital discharges according to patient medical documentation. Moreover, the study collects information about the demographic characteristics of patients and place of residence including residential setting (rural or urban, i.e. residents of cities), according to administrative status of residence area in Poland. The ICD-10 classification, which is officially used in medical documents in Poland, is also applied within NGHMS to determine the codes of the causes of hospital discharges.

#### Mortality

The death registry of Statistics Poland reports underlying causes of death in the Polish population. Information about causes of death is obtained from death certificates, which are issued mandatorily by physicians. Data are partly verified by the regional coding physicians employed by Statistics Poland who determine the ICD-10 code of the underlying cause of the death based on information provided in death certificate [16].

### Case definition and clinical characteristics

The PIDSS does not use a case definition for chickenpox [17]. Physicians report cases based on the clinical course of the disease including the appearance of characteristic symptoms: fever and a general unwell feeling initially, followed by development of a rash that turns into itchy, fluid-filled blisters. Upon onset, the rash covers the trunk, then appears also on the face and scalp, and rarely on the limbs or mucous membranes.

### Statistical analysis

Taking into consideration the distribution of incidences of chickenpox and data aggregation within PIDSS and NGHMS, we calculated incidence and hospital discharge rates for the following age groups: 0–4, 5–9, 10–14, 15–24, 25–64, ≥ 65 years. For those age groups, incidence and hospital discharge rates were calculated for the years 2006–21. For hospital morbidity, we estimated average length of stay. Standardised hospital discharge rates were calculated using the standard of European Union (EU) Standard Population from EUROSTAT [18]. Data on the number of deaths in years 2006–21 according to age groups were imported from Statistics Poland. For hospital morbidity and mortality statistics, the following ICD-10 codes were used for analyses: B01–varicella, B01.0 and B01.1–varicella meningitis and encephalitis, B01.2–varicella pneumonia and B01.8–varicella with other complication. The code B01.9–varicella without complication was excluded, because this code usually is applied in the case of outbreaks of chickenpox in hospitals. To perform all calculations mentioned above, Statistics Poland demographic data were used. The lack of a common unique patient identifier in the dataset precluded cross-linkage between different data sources.

A joinpoint regression model was employed to analyse long-term trends in annual hospital discharge morbidity rates [19] using Joinpoint Trend Analysis Software (version 4.9.1.0) from the National Cancer Institute (NCI), part of the National Institutes of Health (NIH), in the United States. The years 2020 and 2021 were excluded from the joinpoint model because of the limited transmission of the VZV virus as a result of COVID-19 pandemic restrictions, which led to a substantial decline in chickenpox incidences in Poland [20,21].

## Results

### Incidence

During analysed years 2006–21, PIDSS reported 2,516,975 cases of chickenpox in total, of which 16,446 cases were hospitalised with chickenpox without specifying the exact cause of hospital admission. Sex distribution of chickenpox incidence was almost equal: 51.1% of cases were reported in males (n = 1,287,150) and 48.9% in females (n = 1,229,825), respectively. Chickenpox mainly affected children and adolescents up to 14 years, which accounted for 91.8% of reported cases (n = 2,311,206).

**Figure 1:**
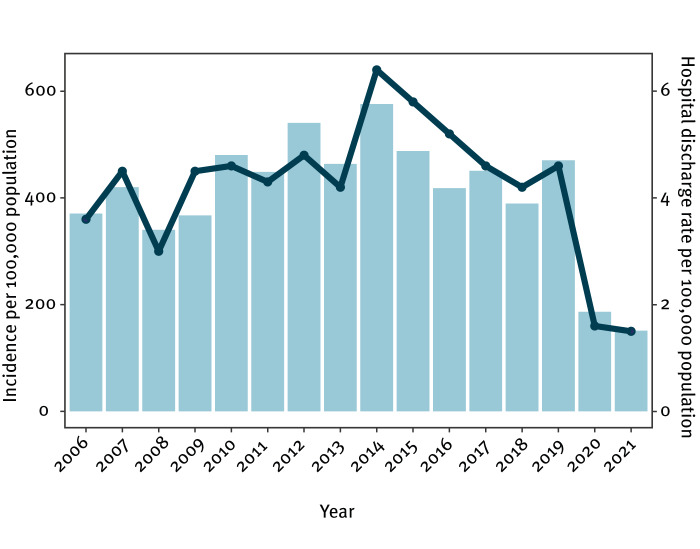
Incidence rates of chickenpox per 100,000 population^a^ and hospital discharge rates^b^ from chickenpox complications, Poland, 2006–2021

^a^ Data extracted from the Polish Infectious Diseases Surveillance System are represented by the bars (n = 2,516,975).

^b^ Data extracted from the Nationwide General Hospital Morbidity Study (ICD-10 codes: B01, B0.1.0, B01.1, B01.2 and B01.8) are represented by the line (n = 25,804).

Data from the PIDSS between 2006 and 2019 indicated a high endemic level of chickenpox compared with the previous 5 years (2000–05), during which the average incidence rate was 315.5 per 100,000 population (range: 234.9–387.8) [22]. In the years 2006–19, the incidence rate of chickenpox ranged from 340.2 to 575.9 per 100,000 population. The highest incidence rate was observed in 2014, and the lowest in 2021. The COVID-19 pandemic led to a sharp decline in incidence, falling below 200 per 100,000 population (Figure 1). 

### Hospital morbidity

In the same years 2006–21, NGHMS registered 33,908 cases hospitalised because of chickenpox. Of these, 25,804 (76%) were related to complications of chickenpox (ICD-10 codes: B01, B0.1.0, B01.1, B01.2, B01.8) and 8,104 (24%) had an uncomplicated course of disease (B01.9). The compiled data from NGHMS and PIDSS revealed that the number of cases requiring hospital treatment because of complications equates to 1% of all reported chickenpox cases (25,804/2,516,975). In respective age groups, an especially high rate of 5.8% hospitalisation (n = 142) was seen in the population 65 years and older, while children aged 5–14 years accounted for less than 1% of hospitalisations with chickenpox complications (n = 8,534). No differences in rates were observed for residents of urban and rural areas (Table 1).

**Table 1:** Accumulated number of hospital discharges because of chickenpox complications^a^ (n = 25,804) and cumulative number of chickenpox cases^b^ (n = 2,516,975) by age group, Poland, 2006–2021

Variables	Age (years)						
	All	0–4	5–9	10–14	15–24	25–64	≥ 65
Total							
Hospital discharges because of chickenpox complications (n)	25,804	12,852	6,757	1,777	1,435	2,841	142
Reported chickenpox cases (n)	2,516,975	1,084,452	1,007,182	219,572	86,473	116,590	2,436
Per cent of cases requiring hospital treatment because of complications	1.0%	1.2%	0.7%	0.8%	1.7%	2.4%	5.8%
Males							
Hospital discharges because of chickenpox complications (n)	13,851	6,935	3,718	940	752	1,445	61
Reported chickenpox cases (n)	1,287,150	551,912	521,478	111,852	42,864	57,757	1,017
Per cent of cases requiring hospital treatment because of complications	1.1%	1.3%	0.7%	0.8%	1.8%	2.5%	6.0%
Females							
Hospital discharges because of chickenpox complications (n)	11,953	5,917	3,039	837	683	1,396	81
Reported chickenpox cases (n)	1,229,825	532,540	485,704	107,720	43,609	58,833	1,419
Per cent of cases requiring hospital treatment because of complications	1.0%	1.1%	0.6%	0.8%	1.6%	2.4%	5.7%
Urban areas							
Hospital discharges because of chickenpox complications (n)	15,487	7,809	3,998	918	796	1,875	91
Reported chickenpox cases (n)	1,483,382	702,560	564,545	100,178	43,728	70,954	1,417
Per cent of cases requiring hospital treatment because of complications	1.0%	1.1%	0.7%	0.9%	1.8%	2.6%	6.4%
Rural areas							
Hospital discharges because of chickenpox complications (n)	10,317	5,043	2,759	859	639	966	51
Reported chickenpox cases (n)	1,033,593	38,1892	442,907	119,394	42,745	45,636	1,016
Per cent of cases requiring hospital treatment because of complications	1.0%	1.3%	0.6%	0.7%	1.5%	2.1%	5.0%

^a^ Data extracted from the Nationwide General Hospital Morbidity Study (ICD-10 codes: B01, B0.1.0, B01.1, B01.2 and B01.8).

^b^ Data extracted from the Polish Infectious Diseases Surveillance System.

Some totals are not equal to the sum of the respective age group sums on account of missing data within reports to the surveillance system. However, these missing data comprise less than 5% of the total.

The specific causes of complications could not be discerned entirely from the ICD-10 codes from hospital discharges between 2006 and 2021, with roughly 96% (n = 24,642) of cases given codes either directly related to chickenpox (B01) or its complications (B01.8). Codes for varicella meningitis or encephalitis (B01.0 and B01.1) and varicella pneumonia (B01.2) were assigned to 2.7% (n = 703) and 1.8% (n = 459) of hospital stays, respectively. The hospitalisation rate per 100,000 population varied over time from 6.4 in 2014 to 1.5 in 2021 , and strong correlation was observed between hospital discharges and incidence rates (R^2^ = 0.92) (Figure 1). The frequency of hospital stays because of complications was at its lowest during COVID-19 pandemic in 2020 and 2021, in line with the substantial decrease in registered incidence rates.

**Figure 2:**
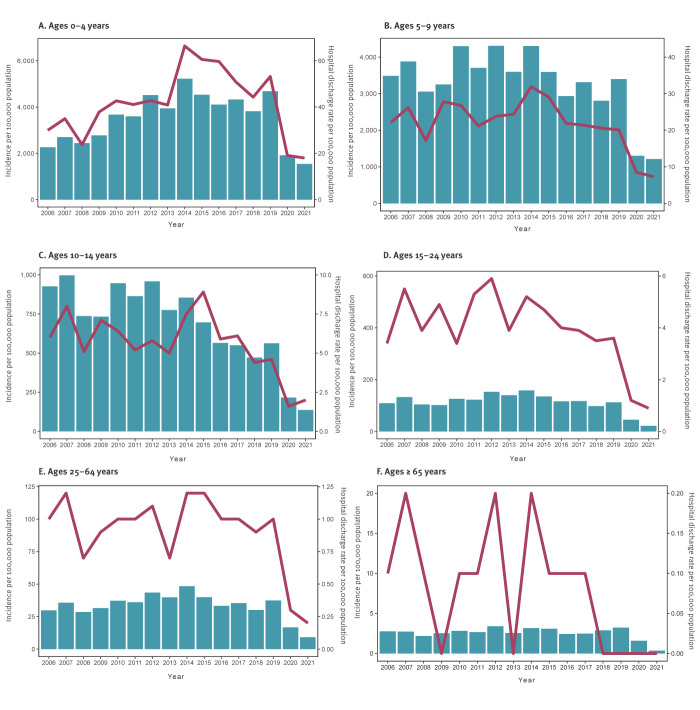
Age-specific incidence rates of chickenpox per 100,000 population^a^ and hospital discharge rates because of chickenpox complications^b^, Poland, 2006–2021

^a^ Data extracted from the Polish Infectious Diseases Surveillance System are represented by the bars.

^b^ Data extracted from the Nationwide General Hospital Morbidity Study (ICD-10 codes: B01, B0.1.0, B01.1, B01.2 and B01.8) are represented by the lines.

An analysis based on sex showed negligible differences in hospitalisation rates because of complications. After age standardisation, the overall hospital discharge rates varied between 2.3 and 9.6 per 100,000 population during the study period. Moreover, there was a noticeable decrease in the average duration of hospital stays because of chickenpox, dropping from 6.4 days in 2006 to 5.0 days in 2021. The highest frequency of hospitalisation was observed among infants and young children up to 4 years old, with a discernible decrease with increasing age. In comparison, in people aged 65 years and above, the data showed only sporadic instances of hospital discharges each analysed year (Figure 2).

The used joinpoint model indicated that no statistically significant trends were observed in the overall crude hospitalisation for complications of chickenpox between 2006 and 2019 (Table 2). However, a notable upward trend was seen in the hospital discharge rates for children aged 0–4 years (average percent change (APC): 4.9%), as well as among the rural population (APC: 3.4%).

**Table 2:** Average percentage change of crude hospital discharge rates because of chickenpox complications per 100,000 population^a^, as determined by joinpoint regression estimations, Poland, 2006–2019^b^

Group	APC	
Overall	2.0	−0.6 to 4.6
Sex		
Males	2.4	−0.1 to 4.9
Females	1.6	−1.2 to 4.5
Age group (years)^c^		
0–4	4.9	1.9 to 8.0*
5–9	−0.5	−3.0 to 2.2
10–14	−1.2	−4.3 to 2.0
15–24	−2.2	−6.0 to 1.7
25–64	0.2	−2.2 to 2,6
Setting of residence		
Urban areas	1.1	−1.4 to 3.7
Rural areas	3.4	0.6 to 6.3*

APC: average percentage change.

^a^ Data Nationwide General Hospital Morbidity Study ICD-10 codes: B01, B0.1.0, B01.1, B01.2 and B01.8.

^b^ The years 2020 and 2021 were excluded from the analysis because of a significant decrease of chickenpox incidences during the COVID-19 pandemic causing atypical disease spread patterns.

^c^ Given the low annual number of hospitalisations in the age group 65 years and older, it was not feasible to estimate APC.

P values < 0.05 are considered significant and are indicated with an asterisk (*).

### Mortality

**Figure 3:**
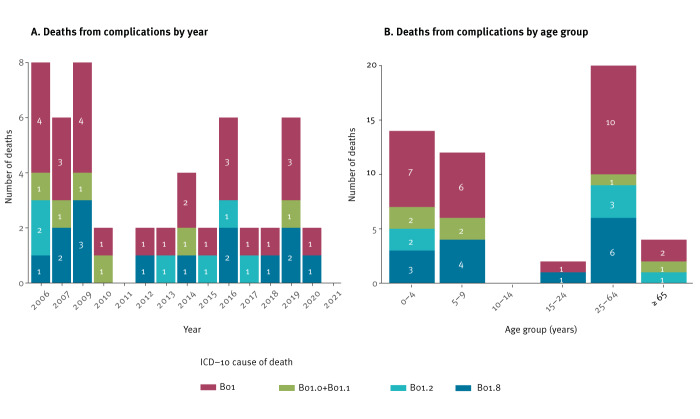
Number of deaths from chickenpox complications as an underlying cause of death, delineated by year and age group, Poland, 2006–2021 (n = 52)

From 2006 to 2021, a total of 52 deaths were reported as being directly linked to complications from chickenpox (n = 34 males and 18 females). Half of all deaths (n = 26) occurred in children aged up to 9 years. Notably, there were no reported deaths in the adolescent age group 10–14 years. Two deaths occurred in age group 15–24 years and 24 among people 25 years and older (Figure 3). Statistics Poland only provides an underlying cause of death and thus it was not possible to identify other existing comorbidities which could have contributed to death. Considering reported cases by PIDSS, the overall case fatality ratio (CFR) for chickenpox during these years was calculated at 0.002%. The age group 65 years and older had the highest fatality ratio at 0.16%.

Data were extracted from death registry of Statistics Poland, ICD-10 codes describing underlying cause of death: B01, B0.1.0, B01.1, B01.2 and B01.8.

The number of deaths attributed to each category of ICD-10 codes describing the underlying cause of death are indicated within individual bars.

The structure of mortality data indicates that, in the majority of the cases, the specific complication leading to death could not be determined. Of the reported deaths, 40 were attributed to chickenpox or related complications (ICD-10 codes B01 and B01.8). A smaller proportion of deaths resulted from varicella pneumonia and varicella meningitis or encephalitis, accounting for six deaths each. The peak years for reported deaths from chickenpox complications were 2006 and 2009, with eight deaths in each of these years (Figure 3).

## Discussion

Our analysis revealed that the risk of chickenpox complications requiring hospitalisation in Poland could potentially be higher than those observed in other European Union (EU) countries. However, the Nationwide General Hospital Morbidity Study does not have datasets for 2022 available yet and therefore we are not able to measure post-pandemic change. Outcomes of NGHMS showed that number of registered hospitalisations from chickenpox including both complicated and uncomplicated course was over 48% higher than number reported within PIDSS in years 2006–21 (n = 33,908 vs n = 16,446). According to 2015 estimations from the European Centre for Disease Prevention and Control (ECDC), standardised hospital discharge rates from chickenpox complications varied from 1.9 to 5.8 per 100,000 population across European countries [23]. In contrast, the standardised rate in Poland during the years 2006–21 varied between 2.3 and 9.6 per 100,000 population. A 2017 study estimating the burden of chickenpox in the EU reported that 0.33% of chickenpox cases required hospital treatment [24]. Our findings reveal that the percentage of complicated chickenpox cases requiring hospitalisation in Poland was significantly higher, at 1%. Furthermore, a study conducted between 2004 and 2017 in England, which used hospital data, demonstrated that the mean annual hospitalisation rate from chickenpox was 8.9 per 100,000 population, with the majority of cases (62%) being related to the course of the disease without complication [2]. In the same study, the rate of hospitalisation because of complications was 3.4 per 100,000 population. In Poland, a much higher percentage of chickenpox hospitalisations (76%) were associated with a complicated course of the disease. 

Our observations support a recommendation to intensify immunisation against chickenpox in Poland. Universal vaccination programmes can reduce the number of chickenpox complications by over 95% and can nearly eliminate hospitalisation as a result of a complicated course of this disease [6,7]. In Poland, vaccines are free of charge and vaccinations are obligatory for children up to 12 years of age who are particularly vulnerable to infection and for children in their vicinity. The estimated maximum vaccine coverage was only up to 2.3% of the total Polish population in 2021.

There are concerns about increasing trends in hospitalisation rates for chickenpox complications among children 0–4 years, and among inhabitants of rural areas. Moreover, deaths with chickenpox as the underlying cause continue to be registered. This may be also due to factors such as declining access to primary care, poorer awareness among parents about chickenpox, or decreasing performance of primary care. However, there is no sufficient evidence to detect and determine the potential impact of these factors on the processes in the Polish healthcare system. Observed trends and still low vaccine coverage suggest that immunisation against chickenpox should be not only intensified but also supported by effective health education of parents and improvements in primary care [25,26]. In addition, understanding attitudes of the Polish society towards varicella vaccination is important to better implement immunisation and build proper strategies to improve health literacy. 

Care must be taken in interpreting results obtained from the study. Previous research has indicated that the number of reported cases of infectious diseases could be lower than the actual incidence [27]. Therefore, under-reporting of chickenpox cases by PIDSS could result in an overestimation of both the fatality rate and the percentage of cases requiring hospitalisation in Poland, relative to other EU countries. It should be also taken into consideration that a proportion of the hospital discharges attributed to chickenpox without complication (B01.9) could have a complicated course. It is worth adding that observed higher hospital discharge rates because of chickenpox were also related to higher chickenpox incidence registered by PIDSS during analysed years in Poland.

Given the lack of a unique identifier, NGHMS did not allow the examination of any potential effect of short-term hospital readmissions of the same patients because of the same causes, which could contribute to registered higher hospital discharges rates. However, short-term re-hospitalisations in general are undesirable and are a limited phenomenon within the healthcare system, affecting mainly people with multiple chronic conditions [28]. Moreover, hospital morbidity can vary significantly between countries because of differences in healthcare system organisation and other socioeconomic factors. It should be noted that hospital morbidity is not as universally comparable in international contexts, as is mortality [15]. It could be also more useful in the future to collect mortality statistics on other comorbidities which may have contributed to death, rather than only underlying causes of deaths. 

## Conclusions

In the years 2006–21, the burden of hospital complications because of chickenpox in Poland seemed to be higher than the average reported by other EU countries. Most of the complications occurred among children under 9 years old, the age group with the highest frequency of chickenpox infections. It may be valuable to consider the intensification of immunisation efforts, supported by health education and primary care improvements. The introduction of universal immunisation program could nearly eliminate deaths from chickenpox and considerably limit the number of hospitalisations because of the disease. It can be useful in the future to merge epidemiological surveillance data with data from different systems collecting data on health situation to evaluate burden scale of complications caused by infectious diseases.
